# Hybrid Matters: Art and Science as a New Epistemology

**DOI:** 10.1089/dna.2021.0527

**Published:** 2022-01-12

**Authors:** Darya Warner

**Affiliations:** ^1^ Department of English and Fine Arts, US Air Force Academy, Colorado, USA.; ^2^ Department of Art, The State University of New York at Buffalo, Buffalo, New York, USA.

**Keywords:** Bioart, ethics, art, science, collaborations, matter

## Abstract

Why do art and science collaborations matter? Hybrid Matters outlines an epistemological mapping of art and science as an emerging method of interrogation. Through the prism of COVID-19, Warner proposes a paradigm shift driven by care and empathy. In opposition to the notion of art as a vehicle for communicating science, Warner suggests a model in which art and science become mutually reinforcing, discovering alternative pathways of understanding in our relationship with natural ecology and one another.

The collaborative practices of art and science present an unprecedented opportunity to generate new forms of knowledge. The artistic and scientific minds are not as divided as we might think—they are fluid interchangeable elements feeding upon each other in a powerful fusion driven by the desire for exploration. The rise of Bioart in the late 20th century is a logical outcome of systems theory ideas, where the concept of emergence reflects rapidly changing social, scientific, technological, and ecological contexts (Capra and Luisi, 2014). Bioart operates in the liminal space between these modes of understanding, fore fronting the organic processes that bind us to all living processes. In this way, Bioart is like a three-dimensional fungal network reaching out to all spheres of life, searching for uncharted epistemological mapping.

Art and science collaborations as an unconventional way of introducing the public to complex science achievements and ideas through empathetic connection created through art.

Working with scientists for the past 10 years, I have discovered that an artist has the potential to spark creative insight in scientists. The notion of “play” and unlimited curiosity vital to artistic creativity works as a catalyst in conversations with scientists, igniting new pathways in scientific research. However, art functions as a platform for scientific exploration where lateral exploration utilizes nonlinear observational techniques resulting in unexpected connections. In this way, the process starts not only from one single point (hypothesis or observation) but also from multiple points simultaneously.

Inspiration and observation emerge in different directions, branching and making connections at unexpected intersections. It is an organic and living process that results in synthesis. The outcome is an artwork (an object, a performance, a concept)—living or nonliving or hybrid that serves as a channel to communicate complex scientific ideas to the public through emotive experience embedded in art. However, how to connect these disciplines? Perhaps the biophilia hypothesis is a framework that can serve as a bridge connecting art and science.

Biophilia, Collaborative Practices, and Radical Care.

The concept of “love of life” was summarized by Aristotle and has been proposed and redefined many times over by others. The term “biophilia” was first coined by Erich Fromm to describe a psychological orientation of being attracted to all that is alive and vital—“love of life or living systems.” Wilson (2006) used the term in the same sense when he suggested that biophilia describes “inborn affinity human beings have for other forms of life an affiliation evoked, according to circumstances, by pleasure, or a sense of security, or awe, or even fascination blended with revulsion” (or “the connections that human beings subconsciously seek with the rest of life.” The possibility of deep connections humans have with other life forms and nature is rooted in our biology.

Since we all originated from common ancestors some billion years ago, Krčmářová (2009), ecologist, anthropologist, and environmental historian at Czech Academy of Sciences, suggests that: “In the biophilia hypothesis, Wilson indicates that the phylogeny of life on Earth is reflected in the structure of the human mind. In his opinion, the human mind must be looked at as one of the parts of the biosphere developing in mutual correlation with its individual elements. In this way, the history of life on Earth is projected into our understanding of the environment and the perception of our existence” (Krčmářová, 2009).

In this sense, biophilia establishes a conceptual bridge between living matter and humans, and art becomes a meeting ground for this connection. One way we might think of framing this is through collaborative practices and the notion of care.

One of the most outstanding examples of collaborative practice is the work of Aganetha Dyck with bees. At first, when she started working with bees, the artist did not intend a collaboration but *used* the bees for their sculptural talent (Taylor, 2013). However, upon extending her research on bees, Dyck experienced a shift toward a more caring practice.

I had experienced this connection working with fungi while growing them in the form of living sculpture in 2016. However, since collaboration requires consent, it poses an unsolvable issue—is there a way to obtain consent from living organisms? In my practice, I developed a framework that helps to examine the notion of living matter and “consent.” *It is Care*. Providing a caring, optimal environment for collaborative projects with organisms can potentially bring us closer to the notion of “consent” with living matter.

For the past 30 years, we have been experiencing a drastic change in our vision of the interaction with nature. “As aesthetic manifestations of posthumanism, Dyck's interspecies collaborations are part of a larger ideological shift from anthropocentrism to eco-mindfulness—a change that disrupts 30,000 years of animal representation in human art” (Taylor, 2013).

The paradigm shift has now been extrapolated into other kinds of living matter. News of plants being able to hear while they are being eaten and bacteria having “latent feelings” has been circulating in mainstream media for over a decade (Bruni *et al*., 2017). Whether these claims are actually based on scientific data or not, it is obvious that our perception of systems of intelligence is changing toward becoming more inclusive of both living and nonliving agents. Stamets (2008), a world-renowned mycologist, believes that mycelium is a fungi's collective consciousness, a network that is beyond the complexity of our most powerful supercomputers. Stamets (2008) postulates that mycelium has been manipulating its environment for billions of years and symbiotically partnered with plants to provide the vital nutrient exchange and information, ensuring plant kingdom survival.

Toshiyuki *et al*. (2004) from Hokkaido University introduced the idea of cellular intelligence in early 2000 by studying slime mold behavior. His study laid a foundation for further recent studies in cellular intelligence, fungi, in particular. Artists such as Heather Barnett, Cesar Baio, and Lucy H.G. Solomon have been working with slime mold in conjunction with collective human intelligence for quite some time now (Barnett, 2018; Baio and Solomon, 2018). There is no doubt that the tendency to redefine the systems of intelligence has been expanding into nonhuman living organisms within the past decades.

However, there is also evidence that this concept is migrating even further into nonliving systems, rethinking the Cartesian split between mind, matter, self, and others through notions of emergence and ecology. In complex systems, *emergence is* a spontaneous order, self-organization (Capra and Luisi, 2014). This concept is seen not only in living systems but also through all the aspects of life and nonlife: nature forces, nonliving physical, and human systems (economics, architecture, artificial intelligence, and language, to name a few).

The distinction between living and nonliving matter and the concept of vitalism* dominated science and philosophy until the late 20th century. However, how does the nonliving become living, and what is a nonliving matter after all?

If we try to assume for a moment that all matter is vital: storms, metals, commodities, and edibles possess a capacity vital materiality, would it be possible to evoke the broader spectrum of biophilia, a “*matterphilia*,” that can encompass everything. In her book “Vibrant Matter,” Bennett (2010) poses a very peculiar question: “Why advocate vibrant matter? Because the image of dead or thoroughly instrumentalized matter feeds human hubris and our EARTH DESTROYING FANTASIES OF CONQUEST AND CONSUMPTION.” Perhaps the introduction of the vibrant matter concept has the potential to shift our anthropocentric approach to the world, decolonize our rigidly hierarchical assumptions and agendas, and create greener forms of human culture fueled by art and science collaborations.

These concepts are the results of art and science collaborative practices that have the potential of creating new systems of knowledge, expanding our understanding of the world around us, and building new social, ecological, and technological connections. Art opens up new pathways for different forms of knowing, and science discovers information through technology. Together, a unique way of knowing can emerge, where patriarchal, colonial, and human-centered mentalities become decentered, and the process of discovery itself becomes a site of exploration of new hybrid space.

Hybrid spaces and entities provide new platforms for possible “*intermatter*” communications and could become the ideal landing spot for various forms of matter to collide and fruit with new kinds of matter. This delicate dance of transformation needs to be based on genuine care, perhaps the type of care we have yet to discover within ourselves—a radical one, care for all the matter.

Art and science collaborations during COVID-19: challenges to overcome, online collaborative connections to explore.

When the COVID-19 pandemic started, my world collapsed like everyone else's. For a while, I did not know how to navigate these new waters of fear and uncertainty. As an artist, I spend a lot of time in solitude but the nature of my work required access to laboratories, which during the pandemic turned out to be impossible. So, I moved my studio to my home and set up a simple home laboratory to continue to work on my thesis. The time that was used for commuting was utilized to build online connections with various laboratories around the world. Within a few months, online exhibits started to pop up, conferences resumed, and Zoom talks became the modus operandi.

I must admit this was an ideal situation for my practice—I was suddenly connected with scientists and artists worldwide—sharing knowledge and insights. Of course, Zoom talks cannot replace live interaction in both art and science. But in my opinion, the pandemic gave me time to stop and rethink my research approach. The isolation made me value human connection even further, but it also created a quiet space within me that was always filled with art shows, openings, and other social activities. The fear of missing something important (the next talk, the next show, the next gathering) was replaced with a much-needed feeling of stillness where I was able to mine new connections to examine my practice and my connection to nature critically.

COVID-19 pandemic provides a circumstance where the fragility of our systems and their interrelationship are revealed in stark relief. By redefining our connection to the world, both human and nonhuman, during pandemic we have inoculated new seeds of art and science collaborations with new possible pathways to humanize our relationships, to inject notions of empathy and care into our relationships, as well as interspecies understanding.

## Notes

^*^
“Vitalism, school of scientific thought—the germ of which dates from Aristotle—that attempts (in opposition to mechanism and organicism) to explain the nature of life as resulting from a vital force peculiar to living organisms and different from all other forces found outside living things. This force is held to control form and development and to direct the activities of the organism” (The Editors of Encyclopaedia Britannica, 2016).
